# Engineering the Image Representation for Deep Learning in Contrast-Enhanced Mammography: A Systematic Analysis of Preprocessing and Anatomical Masking

**DOI:** 10.3390/bioengineering13030322

**Published:** 2026-03-11

**Authors:** Roberta Fusco, Vincenza Granata, Paolo Vallone, Teresa Petrosino, Maria Daniela Iasevoli, Mauro Mattace Raso, Davide Pupo, Piero Trovato, Igino Simonetti, Paolo Pariante, Vincenzo Cerciello, Gerardo Ferrara, Modesta Longobucco, Giulia Capuano, Roberto Morcavallo, Caterina Todisco, Fabiana Antenucci, Mario Sansone, Daniele La Forgia, Antonella Petrillo

**Affiliations:** 1 Radiology Division, Istituto Nazionale Tumori-IRCCS-Fondazione G. Pascale, 80131 Naples, Italy; r.fusco@istitutotumori.na.it (R.F.); p.vallone@istitutotumori.na.it (P.V.); t.petrosino@istitutotumori.na.it (T.P.); m.iasevoli@istitutotumori.na.it (M.D.I.); m.mattaceraso@istitutotumori.na.it (M.M.R.); davide.pupo@istitutotumori.na.it (D.P.); piero.trovato@istitutotumori.na.it (P.T.); igino.simonetti@istitutotumori.na.it (I.S.); p.pariante@istitutotumori.na.it (P.P.); a.petrillo@istitutotumori.na.it (A.P.); 2 Division of Health Physics, Istituto Nazionale Tumori-IRCCS-Fondazione G. Pascale, IRCCS di Napoli, 80131 Naples, Italy; v.cerciello@istitutotumori.na.it; 3 Pathology Division, Istituto Nazionale Tumori-IRCCS-Fondazione G. Pascale, 80131 Naples, Italy; gerardo.ferrara@istitutotumori.na.it; 4 Struttura Semplice Dipartimentale di Radiodiagnostica Senologica, IRCCS Istituto Tumori Giovanni Paolo II, Via Orazio Flacco 65, 70124 Bari, Italy; m.longobucco@oncologico.bari.it (M.L.); g.capuano@oncologico.bari.it (G.C.); r.morcavallo@oncologico.bari.it (R.M.); todisco.katia@gmail.com (C.T.); d.laforgia@oncologico.bari.it (D.L.F.); 5 Biomedical Engineering Faculty, Università degli Studi di Napoli Federico II, 80125 Naples, Italy; f.antenucci@studenti.unina.it (F.A.); msansone@unina.it (M.S.)

**Keywords:** oncology, breast, deep learning, convolutional neural networks

## Abstract

Deep-learning models applied to contrast-enhanced mammography (CEM) are known to be highly sensitive to the input image representation. However, preprocessing is often treated as a secondary step and rarely analyzed as an independent design variable. In this work, we present a systematic engineering analysis of a deterministic, label-independent preprocessing pipeline for CEM images. The pipeline integrates intensity normalization, global histogram matching, local contrast enhancement, denoising, and anatomically constrained breast masking. Using a controlled experimental design, identical deep-learning architectures were trained under different input representations to isolate the impact of preprocessing on classification performance and stability. Across convolutional neural network architectures, anatomically constrained preprocessing consistently improves discrimination performance, reduces variability across cross-validation folds, and enhances training stability. Breast mask-based representations demonstrate substantial gains in AUROC and AUPRC compared to raw DICOM inputs. These findings highlight image preprocessing as a first-class engineering component in medical AI pipelines. Breast masking significantly improves robustness and generalization, independently of network architecture complexity. From a clinical perspective, improving model robustness and sensitivity to malignant lesions may contribute to more reliable AI-assisted decision support in contrast-enhanced mammography, particularly in settings characterized by acquisition variability and heterogeneous patient populations.

## 1. Introduction

Contrast-enhanced mammography (CEM) has emerged as a valuable imaging modality for breast cancer assessment, combining high-resolution mammographic imaging with functional information derived from iodinated contrast uptake. By enhancing lesion conspicuity and suppressing background parenchymal tissue, CEM improves sensitivity compared to conventional mammography, particularly in dense breasts [1,2,3,4,5,6,7,8,,9,10,11].

Despite these advantages, CEM images remain affected by substantial variability related to acquisition protocols, scanner vendors, patient anatomy, and contrast dynamics. This variability poses significant challenges for automated image analysis systems, especially deep-learning (DL)-based methods, which are known to be highly sensitive to input data distribution and representation [12,13,14,15,16,17,18,19,20,21,22,23,24,25,26,27,28].

Recent advances in convolutional neural networks (CNNs) and hybrid CNN–Transformer architectures have demonstrated promising results in mammographic image classification and lesion detection [29,30,31,32,33,34,35,36,37,38,39,,40,41,42]. However, most published studies focus on architectural innovations or performance optimization while treating image preprocessing as a fixed or auxiliary step. As a result, the role of preprocessing as an engineering design variable remains underexplored.

From an engineering perspective, preprocessing defines the representation space in which learning takes place. In medical imaging, this representation directly affects signal-to-noise ratio, anatomical consistency, and the balance between diagnostically relevant and irrelevant information. Prior studies have shown that intensity normalization, histogram-based enhancement, and contrast equalization can improve lesion visibility and downstream learning stability in mammography [32,33,34,43]. Nevertheless, these techniques are often evaluated in isolation or embedded within complex pipelines without systematic comparison.

Another critical yet underinvestigated aspect is the explicit incorporation of anatomical constraints. Breast masking techniques aim to suppress background regions and acquisition artifacts, allowing models to focus exclusively on anatomically meaningful content. Automated breast region segmentation tools, such as the LIBRA framework [7,8], have been widely adopted for quantitative breast imaging and density assessment, but their role as a preprocessing strategy for deep learning remains insufficiently characterized.

The objective of this study is to perform a controlled, engineering-oriented evaluation of image preprocessing strategies for deep learning applied to contrast-enhanced mammography. Rather than proposing a novel classification architecture, we focus on isolating the impact of preprocessing and anatomical masking on model performance, robustness, and stability. By training identical deep-learning models under different input representations, we aim to quantify how preprocessing choices influence downstream learning independently of network design.

The main contributions of this work are as follows:

1. The definition of a deterministic, label-independent preprocessing pipeline for CEM images, designed to harmonize intensity distributions and enhance diagnostically relevant structures.
2. A systematic comparison of raw, enhanced, and anatomically constrained image representations under identical training conditions.
3. An engineering analysis of the effects of breast masking on classification performance, training stability, and generalization.

## 2. Materials and Methods

This study used exclusively the publicly available CDD-CESM dataset hosted by The Cancer Imaging Archive (TCIA) [31,33]. The dataset includes 1003 recombined contrast-enhanced spectral mammography (CESM) images acquired using dual-energy techniques. For each examination, cranio-caudal (CC) and mediolateral oblique (MLO) views were obtained approximately two minutes after intravenous administration of iodinated contrast material (1.5 mL/kg). Each acquisition consists of low-energy (26–31 kVp) and high-energy (45–49 kVp) exposures, which are digitally recombined through subtraction to generate contrast-enhanced images highlighting areas of contrast uptake. The dataset provides annotated images and associated clinical information, enabling supervised learning experiments without the need for additional institutional data. All image preprocessing, anatomical breast masking, and convolutional neural network (CNN) analyses were performed centrally to ensure methodological consistency.

### 2.1. Study Design Overview

This study was designed as a controlled experimental analysis aimed at isolating the effect of image preprocessing on deep-learning-based classification of contrast-enhanced mammography images. All experiments were conducted using identical network architectures, training procedures, data splits, and evaluation metrics, while varying only the input image representation. The methodological workflow followed a structured pipeline consisting of image preprocessing and representation generation, deep-learning model training, and performance evaluation.

Image preprocessing included intensity normalization, contrast harmonization, local enhancement, denoising, and anatomical breast masking. All preprocessing operations were applied globally and independently of class labels to prevent data leakage and preserve the validity of the experimental comparison. All images were first converted to a uniform format and rescaled to a fixed spatial resolution. Pixel intensity normalization was performed using min–max scaling to standardize the dynamic range. Global histogram matching was applied to reduce inter-image contrast variability within the dataset. Local contrast enhancement and denoising were subsequently performed using fixed parameter settings across all images.

All processed images were resized to a unified input dimension compatible with the CNN architectures. Anatomical breast masking was applied using an automated segmentation procedure implemented consistently across the entire dataset. Preprocessing was executed centrally using identical code and parameter configurations for all images. Data splitting into training, validation, and test sets was performed after preprocessing to ensure strict separation between model development and evaluation phases.

A detailed description of each preprocessing step is provided in the following sections.

### 2.2. Image Preprocessing Pipeline

The image preprocessing pipeline was designed to standardize contrast-enhanced mammography images and to explicitly control the input representation provided to deep-learning models. All preprocessing steps were deterministic, label-independent, and applied uniformly to the entire dataset prior to data splitting, ensuring reproducibility and preventing information leakage.

The pipeline consists of five sequential stages: intensity normalization, global histogram matching, local contrast enhancement, denoising, and anatomical breast masking. Each component was selected based on established image processing principles and prior evidence in mammographic analysis [32,33,34,43].

#### 2.2.1. Intensity Normalization and Percentile-Based Windowing

Raw CEM DICOM images exhibit wide intensity variability due to differences in the acquisition machine, breast composition, and contrast agent uptake. To mitigate the influence of extreme intensity outliers and harmonize the dynamic range across images, a percentile-based windowing strategy was adopted.

Specifically, pixel intensities were clipped between the 5th and 99.5th percentiles of the image intensity distribution, following robust normalization strategies previously applied in mammographic image processing [32]. This approach suppresses acquisition artifacts and isolated high-intensity pixels while preserving diagnostically relevant contrast-enhanced structures.

After windowing, intensities were linearly rescaled to the [0, 1] interval and converted to an 8-bit representation. All images were then resized to a fixed spatial resolution to ensure homogeneity of input dimensions across the dataset.

#### 2.2.2. Global Histogram Matching

To further reduce inter-patient and inter-device variability, a global histogram matching (GHM) procedure was applied. Histogram-based normalization has been widely used in mammography to improve contrast consistency and reduce domain shifts between acquisitions [43].

A global reference cumulative distribution function (CDF) was computed from the entire dataset after background exclusion. Each individual image histogram was then transformed to match this reference distribution. Unlike adaptive or local normalization strategies, this global approach enforces consistent intensity statistics across all images while preserving relative contrast relationships within each image.

By standardizing intensity distributions at the dataset level, GHM reduces spurious correlations related to scanner-specific characteristics and facilitates more stable learning in downstream deep-learning models.

#### 2.2.3. Local Contrast Enhancement

Following global normalization, local contrast enhancement was applied to improve the visibility of fine structural details and lesion boundaries. A Local Contrast Mapping (LCM) operator was employed, implemented by subtracting a Gaussian-smoothed version of the image from the normalized image, thereby enhancing high-frequency components.

This operation is conceptually similar to unsharp masking techniques and has been previously reported to improve lesion detectability in mammographic images [33]. By emphasizing local intensity variations, LCM enhances edges and subtle textural patterns that may be informative for classification.

Subsequently, Contrast-Limited Adaptive Histogram Equalization (CLAHE) was applied to further enhance micro-contrast while controlling noise amplification. CLAHE has been extensively adopted in computer-aided detection systems for mammography due to its ability to improve contrast in low-visibility regions without introducing excessive artifacts [33].

#### 2.2.4. Denoising

To reduce noise amplification introduced by local contrast enhancement, a wavelet-based denoising strategy was applied using the denoise_wavelet function from the Scikit-image library (version 0.25.2). Denoising was performed using multilevel discrete wavelet decomposition with a Daubechies wavelet (db1), two decomposition levels, and soft thresholding. The BayesShrink method was employed for threshold estimation, with sigma rescaling enabled. The denoising step was applied after local contrast enhancement and prior to anatomical breast masking, ensuring consistent noise reduction while preserving structural image content relevant for downstream convolutional neural network training.

#### 2.2.5. Anatomical Breast Masking

In addition to intensity-based enhancement, anatomically constrained representation was generated by isolating the breast region from background structures and acquisition artifacts. Automated breast segmentation was performed using the LIBRA framework, a validated tool for breast region extraction released as part of CaPTk [7,8].

LIBRA employs intensity-based filtering, morphological refinement, and anatomical priors to separate breast tissue from the background, pectoral muscle, and non-anatomical elements. The algorithm was applied directly to the original CEM DICOM images to generate a binary breast mask for each mammographic view.

**Figure 1:**
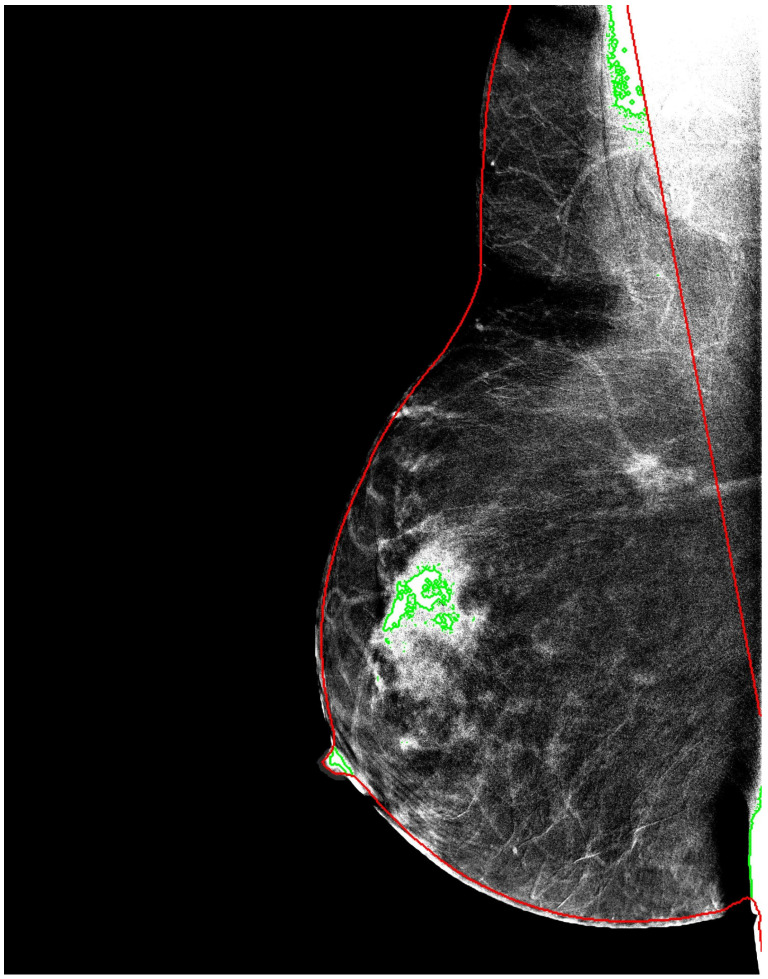
Example of breast segmentation using the LIBRA framework. The red contour outlines the breast boundary after background and pectoral muscle removal, while green areas indicate detected lesions within the anatomically constrained breast region.

The resulting masks were used to create an alternative input representation, referred to as breast-mask-based images, in which all non-breast pixels were suppressed. This representation explicitly encodes anatomical constraints while discarding background information that is unlikely to contribute to lesion characterization (Figure 1).

**Figure 2:**
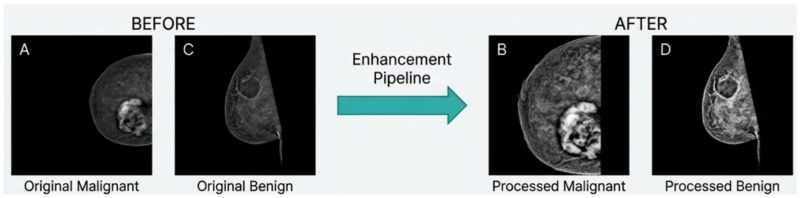
Representative examples of the multi-stage enhancement pipeline. Panels (**A**,**C**) show original malignant and benign CEM images, while panels (**B**,**D**) display the corresponding processed images after normalization and contrast enhancement. The pipeline enhances tissue contrast and structural visibility while preserving lesion morphology.

Unlike lesion-level segmentation, breast masking does not incorporate diagnostic labels and therefore preserves independence between preprocessing and classification. By introducing an explicit anatomical prior at the input level, this strategy reduces the dimensionality of the learning problem and encourages models to focus on anatomically meaningful structures. The Figure 2 reports representative examples of the multi-stage enhancement pipeline.

#### 2.2.6. Preprocessing Integrity and Data Leakage Considerations

All preprocessing operations were implemented as deterministic and label-independent transformations. No class-conditioned statistics, outcome information, or partition-specific parameters were used at any stage of the pipeline.

Percentile-based intensity clipping was applied independently to each image based solely on its internal intensity distribution. Therefore, this step does not introduce cross-sample statistical coupling.

Global histogram matching relied on a single reference cumulative distribution function (CDF) computed across the dataset to standardize intensity statistics. Although this reference distribution was estimated prior to data partitioning, it represents a deterministic normalization transform and does not encode class-specific or patient-specific information. No preprocessing parameter was optimized based on model performance, validation results, or test outcomes.

Data partitioning into training, validation, and test subsets was performed strictly at the patient level after preprocessing, ensuring that no patient contributed images to multiple partitions. While fold-specific estimation of statistical normalization parameters could theoretically reinforce partition independence, the applied transformations are fully label-independent and do not incorporate predictive information. Consequently, meaningful performance inflation due to preprocessing-induced leakage is not expected.

The global histogram reference acts as a fixed-intensity standardization mapping and does not encode class-dependent or predictive information. Therefore, although estimated on the full dataset, it does not introduce outcome-driven leakage nor provide privileged information to the training process.

### 2.3. Deep-Learning Models

To avoid confounding effects related to architectural complexity, this study intentionally focused on a limited set of well-established and widely adopted convolutional neural network (CNN) architectures. The objective was not to maximize classification performance, but to evaluate how different input representations affect learning behavior and robustness when using standard and interpretable deep-learning models.

Two representative CNN architectures were selected: VGG16 and MobileNetV2. These models were chosen to span different computational regimes—high-capacity and lightweight—while remaining conceptually simple and widely used as baseline architectures in medical imaging studies [28,33,37,39]. This selection enables assessing whether the impact of anatomical breast masking persists independently of model capacity and computational complexity.

#### 2.3.1. VGG16

VGG16 is a deep convolutional neural network characterized by a sequential architecture composed of stacked 3 × 3 convolutional layers followed by max-pooling operations and fully connected layers [28]. Despite its relatively large number of parameters, VGG16 remains conceptually simple and provides a strong baseline for image classification tasks.

Its homogeneous design makes VGG16 particularly suitable for studying the effects of input preprocessing, as changes in performance can be more directly attributed to differences in image representation rather than to complex architectural mechanisms.

#### 2.3.2. MobileNetV2

MobileNetV2 is a lightweight convolutional neural network architecture designed for computational efficiency, based on depthwise separable convolutions and inverted residual blocks [39]. This design significantly reduces the number of parameters and computational cost while preserving representational capacity.

Compared to conventional deep CNNs, MobileNetV2 is particularly well suited for deployment-oriented and resource-constrained scenarios, which are increasingly relevant in medical imaging applications. Its inclusion in this study allows evaluating whether the benefits of anatomical breast masking extend to efficient architectures, thereby assessing the generality of representation-driven performance gains beyond high-capacity models.

### 2.4. Training Strategy

The models were trained using an identical supervised learning framework to ensure comparability across experiments. The classification task was formulated as a binary problem, distinguishing malignant from benign/negative cases. Data partitioning was performed strictly at the patient level to prevent information leakage, ensuring that all images belonging to a given patient were assigned exclusively to a single subset. No patient contributed data to more than one fold or to both training and test sets.

Training was performed using cross-entropy loss optimized with the Adam optimizer. A fixed batch size of 32 was employed across all experiments. To mitigate class imbalance, a controlled undersampling strategy was applied to the majority class, ensuring balanced mini-batches during training.

A stratified five-fold cross-validation scheme was adopted at the patient level to assess model robustness and reduce variance related to data partitioning. In each fold, approximately 80% of patients were used for training and internal validation, while 20% were held out for fold-based validation. After cross-validation, model performance was further evaluated on an independent hold-out test set defined prior to training, comprising 15% of malignant patients with balanced sampling of benign/negative cases. The test set remained completely unseen during model development and hyperparameter selection.

Model weights were initialized using ImageNet pretraining, followed by fine-tuning of all layers. Since contrast-enhanced mammography images are inherently grayscale, each preprocessed image was converted to a three-channel format by duplicating the single intensity channel across the RGB dimensions. This step ensured compatibility with convolutional neural networks pretrained on ImageNet, which expect three-channel inputs. Standard ImageNet normalization (mean = [0.485, 0.456, 0.406]; standard deviation = [0.229, 0.224, 0.225]) was subsequently applied without modification. Because all three channels contain identical intensity values, this procedure preserves the intrinsic grayscale structure while aligning the input distribution with pretrained backbone expectations.

To isolate the impact of image representation, data augmentation was intentionally restricted to mild geometric transformations, specifically horizontal flipping and small rotations. More aggressive augmentation strategies were avoided, as they could introduce artificial variability in image geometry or intensity distributions, potentially confounding the representation-level effects under investigation. By maintaining minimal augmentation, the experimental design preserved anatomical realism and ensured that observed performance differences were primarily attributable to input representation rather than synthetic variability.

### 2.5. Experimental Setup

Each CNN architecture was trained under two different input representations, both derived from the same preprocessing pipeline described in Section 2.2. The first representation consists of preprocessed full-field contrast-enhanced mammography images. The second representation is obtained by applying an automatically generated breast mask to the same preprocessed images, thereby retaining only breast tissue and suppressing background regions.

All preprocessing steps, including intensity normalization, contrast enhancement, and denoising, were identical in both conditions.

All other experimental parameters—including network architecture, optimizer, learning rate, batch size, and training schedule—were kept constant. This design ensures that observed performance differences can be attributed exclusively to the effect of anatomical constraint in the input representation.

#### CNN Training Configuration and Computational Environment

All experiments were implemented in Python 3.x using PyTorch (version 2.5.1+cu121) and Torchvision (version 0.20.1+cu121) libraries. Pretrained ImageNet weights were used for all backbone architectures (VGG16 and MobileNetV2). Transfer learning was implemented by initially freezing all convolutional backbone layers and setting their parameters to non-trainable during a five-epoch warm-up phase, during which only the newly initialized final fully connected classification layer was optimized. This strategy allowed the classification head to adapt to the CEM domain while preserving pretrained feature representations. After the warm-up phase, all backbone layers were unfrozen by enabling gradient computation across the entire network, and fine-tuning was performed jointly on all layers using differential learning rates to allow gradual adaptation of pretrained filters.

Optimization was performed using the AdamW optimizer with separate learning rates for backbone and classification layers. The learning rate for the classification head was set to 1 × 10^−4^, while the backbone learning rate was set to 3 × 10^−5^, with a weight decay coefficient of 1 × 10^−4^. Learning rate scheduling was implemented using a Cosine Annealing scheduler with T_max = 20 epochs. Training was conducted for a maximum of 30 epochs per fold, with early stopping applied based on validation performance using a patience of 6 epochs.

The loss function was Cross-Entropy Loss without class weighting. Class imbalance at the case level was addressed through moderate undersampling of the majority class with a ratio of 2.0. All input images were resized to 224 × 224 pixels. During training, light data augmentation was applied, including random rotations within ±10 degrees, random affine transformations with translation up to ±5%, scaling between 0.9 and 1.1, shear up to ±8 degrees, and horizontal flipping with probability 0.5.

Model evaluation was performed using five-fold stratified cross-validation at the patient level, followed by assessment on an independent hold-out test set comprising 15% of malignant patients with balanced benign/negative sampling.

The optimal classification threshold was determined using the Youden index computed on validation predictions within each fold of the five-fold cross-validation. To obtain a single operating point for final evaluation, validation probabilities from all folds were aggregated, and a global threshold was estimated from the combined validation outputs. This threshold was then applied unchanged to the independent hold-out test set. The test data were not used at any stage for threshold optimization.

All numerical operations were performed using NumPy (2.2.6), data handling with Pandas (2.3.3), performance metrics with Scikit-learn (1.7.2), image preprocessing with Scikit-image (0.25.2) and OpenCV (4.12.0.88), DICOM handling with pydicom (3.0.1), EfficientNet implementation via efficientnet_pytorch (0.7.1), visualization using Matplotlib (3.10.7), and progress monitoring with tqdm (4.67.1). GPU acceleration was enabled using CUDA 12.1.

Experiments were conducted on a 64-bit Windows system (build 10.0.22631) equipped with an Intel^®^ Core™ i9-12900 CPU (2.40 GHz), 64 GB RAM, and 6 GB GPU memory. To ensure reproducibility, a fixed random seed (42) was applied to Python, NumPy, and PyTorch operations, and deterministic cuDNN settings were enabled across all runs.

### 2.6. Evaluation Metrics

Model performance was evaluated using a combination of threshold-dependent and threshold-independent metrics commonly recommended for imbalanced binary classification tasks in medical imaging [31,33]. The primary evaluation metric was the Area Under the Receiver Operating Characteristic Curve (AUROC), which quantifies the model’s ability to discriminate between classes independently of the decision threshold. Additionally, the Area Under the Precision–Recall Curve (AUPRC) was reported to provide a more informative assessment under class imbalance.

During cross-validation, AUROC and AUPRC were computed independently for each fold using the predicted probabilities of the validation subset and subsequently averaged across the five folds to estimate robustness and variance. For the independent hold-out test set, AUROC and AUPRC were computed on merged predictions obtained from the final trained model across the entire test cohort, without fold averaging.

To evaluate classification performance at a fixed operating point, Balanced Accuracy—defined as the average of sensitivity and specificity—was computed. Sensitivity (recall for malignant cases) and specificity were also reported separately to characterize error asymmetry.

## 3. Results

When trained on original images, VGG16 achieved an AUROC of 0.815 on the independent test set (Table 1). The confusion matrix analysis shows a specificity of 0.812 and a sensitivity for malignant cases of 0.608, resulting in an overall accuracy and balanced accuracy of 0.705. Training histories across the five cross-validation folds exhibit a consistent pattern: training loss decreases monotonically, whereas validation loss shows oscillations and late increases. Similarly, training accuracy steadily increases above 0.90, while validation accuracy stabilizes around 0.70–0.75, widening the gap between the two curves. This behavior indicates progressive overfitting and limited generalization when learning directly from original images.

For MobileNetV2 trained on original images, ROC analysis on the test set shows an AUROC of 0.732. The corresponding confusion matrix yields a sensitivity of 0.602 for malignant cases and a specificity of 0.739, resulting in an accuracy and balanced accuracy of 0.651. These results indicate moderate discriminative performance, comparable to VGG16, with relatively higher specificity than sensitivity. This pattern suggests that background structures and acquisition-related variability in original images negatively affect generalization, particularly for lightweight architectures.

When the same architectures were trained on breast-mask-based images, a systematic improvement in performance and training stability was observed for both networks (Table 1). For VGG16, breast masking led to a clear increase in discriminative performance. The model achieved an AUROC of 0.872 and a balanced accuracy of 0.774 on the test set. Sensitivity for malignant cases increased to 0.726, while specificity remained high at 0.821. Training curves across all five cross-validation folds show more stable convergence, with reduced divergence between training and validation loss and a smaller gap between training and validation accuracy. This behavior indicates improved generalization compared to training on original images.

Similarly, MobileNetV2 benefited from breast masking. When trained on breast-mask-based images, the model achieved an AUROC of 0.788 and a balanced accuracy of 0.712, with sensitivity for malignant cases increasing to 0.641 while specificity improved to 0.793.

To assess whether these improvements were statistically significant, paired comparisons were conducted between the original and breast-mask-based conditions on the independent test set. AUROC values were compared using DeLong’s test for correlated ROC curves, and classification outcomes were evaluated using McNemar’s test on matched predictions. In addition, fold-level AUROC and balanced accuracy were compared using a paired Wilcoxon signed-rank test across the five cross-validation folds. These analyses confirmed that breast masking significantly improved discrimination performance and reduced misclassification rates (*p* < 0.05 for both architectures).

Overall, these findings confirm that anatomical constraint improves learning behavior for both high-capacity and lightweight models, reducing the negative impact of background regions and enhancing generalization performance when training exclusively on the public CDD-CESM dataset.

**Figure 3:**
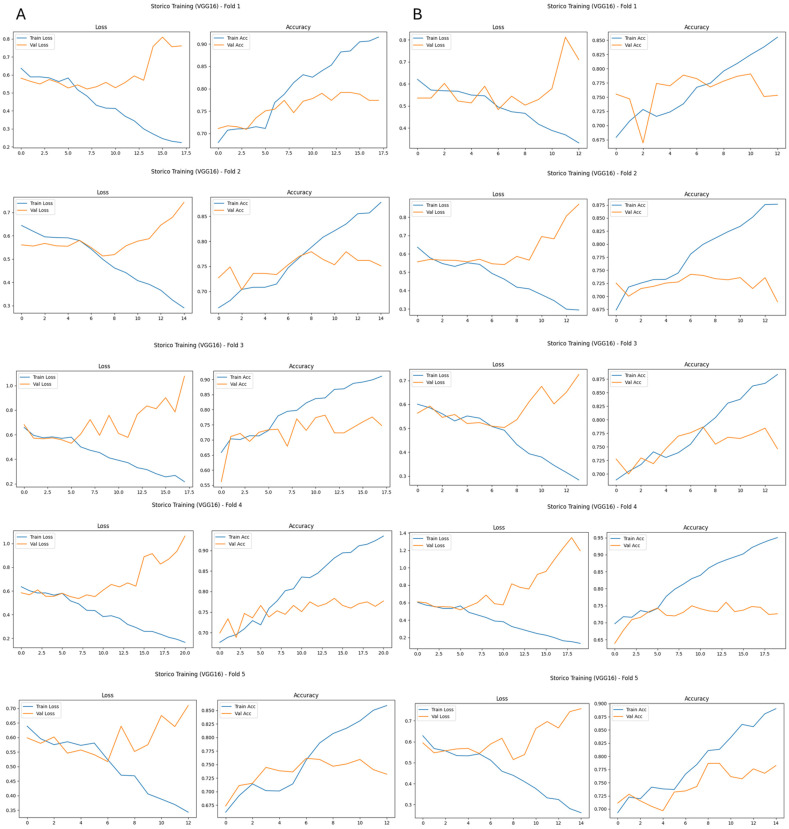
Training history of the VGG16 model across five cross-validation folds. (**A**) Loss and accuracy curves using original images, showing progressive divergence between training and validation performance. (**B**) Loss and accuracy curves using breast-mask-based images, demonstrating more stable convergence and reduced overfitting.

**Figure 4:**
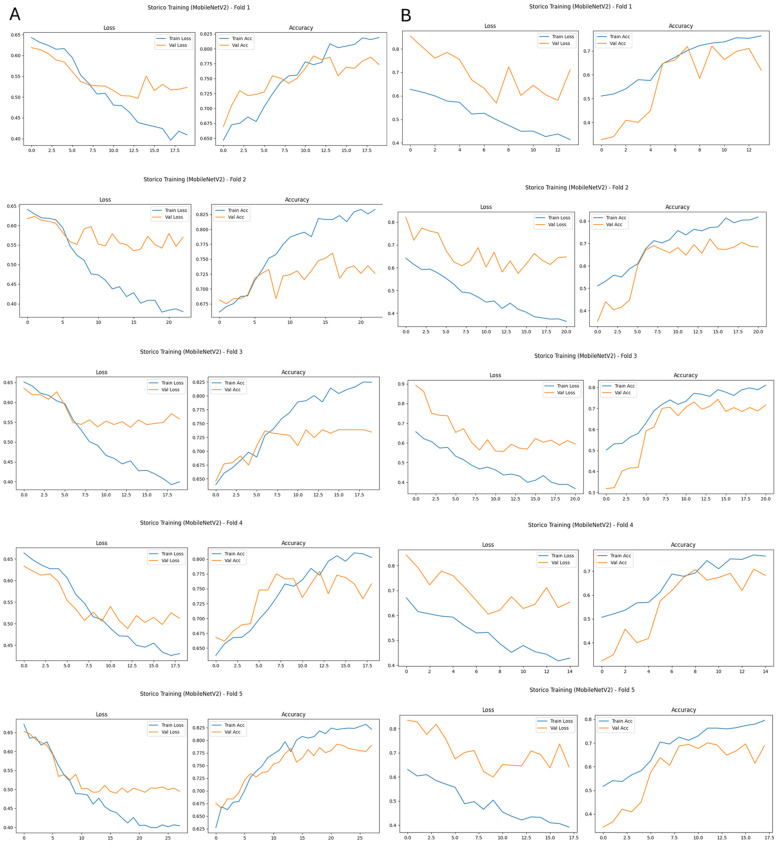
Training history of the MobileNetV2 model across five cross-validation folds. (**A**) Loss and accuracy curves using original images, showing variability across folds and limited generalization. (**B**) Loss and accuracy curves using breast-mask-based images, demonstrating improved stability and more consistent validation performance.

Across both architectures, breast-mask-based images resulted in more consistent training dynamics across cross-validation folds, as illustrated in Figure 3 and Figure 4. For models trained on original images, validation loss and accuracy curves exhibit larger inter-fold variability and clear signs of overfitting, with an increasing divergence between training and validation performance (Figure 3A and Figure 4A).

In contrast, when breast-mask-based images are used, both VGG16 and MobileNetV2 show smoother convergence behavior, reduced variance across folds, and more stable validation performance (Figure 3B and Figure 4B). These trends indicate improved generalization and reduced sensitivity to data partitioning, highlighting the stabilizing effect of anatomical constraint on the learning process.

Overall, the results demonstrate the following:

• Training on original images leads to moderate performance and limited generalization for both VGG16 and MobileNetV2.
• Breast-mask-based representations systematically improve discrimination, sensitivity to malignant cases, and cross-fold training stability.
• The observed improvements are consistent across both a high-capacity model (VGG16) and a lightweight architecture (MobileNetV2), indicating that the effect of anatomical masking is largely independent of model complexity.

The robustness of the preprocessing framework is further supported by the cross-validation design and independent test evaluation. Since all preprocessing transformations were deterministic, label-independent, and applied uniformly across experimental conditions, the relative performance comparison between the original and breast-mask-based representations does not depend on partition-specific statistical estimation. The consistent trends observed across cross-validation folds (Figure 3 and Figure 4) indicate that the reported improvements are not driven by partition-specific statistical artifacts.

## 4. Discussion

In recent years, research in medical imaging has largely focused on architectural innovation, including increasingly complex convolutional and hybrid deep-learning models [13,17,30]. While such approaches have improved benchmark performance, comparatively less attention has been devoted to deterministic preprocessing and input representation design, despite their recognized influence on learning behavior in medical imaging pipelines [14,15,16]. In breast imaging, background variability, acquisition-dependent artifacts, and heterogeneous tissue composition can introduce confounding information that neural networks may inadvertently exploit [7,10,23,24,44,45,46,47,48,49,50]. When preprocessing is not explicitly controlled, models may learn dataset-specific correlations that reduce generalization across folds or institutions, an issue particularly critical in medical imaging, where datasets are smaller and less standardized than natural image benchmarks [15].

Several studies have reported that increasing architectural complexity does not consistently translate into improved robustness or generalization in mammographic classification tasks [26,30]. In contrast, representation-level interventions—such as anatomical masking or region selection strategies—have demonstrated the ability to stabilize training and enhance discrimination performance without altering network topology [18,23,25]. Moreover, preprocessing pipelines are often underreported or insufficiently standardized, limiting reproducibility and cross-study comparability [18,23]. In many CEM studies, lesion-level cropping or ROI extraction guided by radiologist annotations is employed [18,21,24]; although effective, such strategies introduce annotation dependency and may inadvertently couple preprocessing with diagnostic labels. Additionally, preprocessing variations are frequently combined with architectural modifications or attention mechanisms [26,30], making it difficult to isolate the specific contribution of input representation. Limited emphasis has also been placed on training stability and cross-fold variability, despite the known susceptibility of medical imaging datasets to overfitting and domain variability [15].

The present study addresses these methodological gaps through a controlled, deterministic, and label-independent preprocessing framework. By maintaining identical training conditions and network architectures while modifying only the input representation, we explicitly isolate the impact of anatomical breast masking on discrimination performance and training stability.

Models trained on original contrast-enhanced mammography images achieved moderate discriminative performance but exhibited reduced sensitivity to malignant lesions and signs of overfitting, as reflected by a divergence between the training and validation curves. This behavior aligns with previous observations showing that background structures and acquisition variability can introduce confounding signals during model training [7,9,23,24], particularly in relatively small datasets [15,20].

The introduction of anatomical breast masking produced consistent improvements across all evaluated metrics and stabilized training dynamics. Both VGG16 and MobileNetV2 demonstrated higher AUROC and AUPRC values, increased sensitivity to malignant cases, and improved balanced accuracy. Training convergence became smoother, with reduced inter-fold variability. From an engineering perspective, breast masking can be interpreted as the incorporation of an explicit anatomical prior at the input level, constraining learning to clinically meaningful regions without requiring lesion-level annotations. The consistency of performance improvements across cross-validation folds and on the independent test set further supports that the observed gains are attributable to anatomical masking rather than to partition-specific statistical effects. An alternative interpretation of the observed performance gains is that breast masking reduces confounding background signals, such as image borders, acquisition artifacts, textual markers, or peripheral noise, rather than exclusively enhancing anatomical focus. This explanation is plausible and does not contradict the proposed mechanism. From a machine-learning perspective, removing irrelevant background structures reduces spurious correlations and limits the possibility that the model exploits non-diagnostic cues. Therefore, the observed improvements may result from a combined effect of anatomical constraint and suppression of acquisition-related confounders. Both mechanisms contribute to improved representation quality and training stability.

Similar strategies have been shown to enhance robustness and reduce overfitting in breast imaging tasks [7,15,23], and suppression of irrelevant background information has previously been associated with improved classification performance in CEM and MRI studies [10,24,25,44,45,46].

Importantly, these improvements were observed across architectures with substantially different computational profiles. VGG16, a high-capacity convolutional network [28], and MobileNetV2, a lightweight, efficiency-oriented architecture [39], both exhibited comparable relative gains when trained on anatomically constrained images. This consistency suggests that the impact of anatomical masking is largely independent of model complexity. Prior comparative analyses have indicated that architectural sophistication alone does not guarantee improved generalization when input variability remains uncontrolled [26,30]; our findings reinforce the central role of representation design in shaping learning behavior in data-limited medical imaging settings.

Preprocessing should therefore be regarded as a core engineering component of medical AI pipelines rather than a secondary implementation detail. By improving input quality instead of increasing model complexity, it is possible to enhance robustness and reproducibility without incurring additional computational cost. This is particularly relevant for deployment-oriented models, where lightweight architectures such as MobileNetV2 are preferred for scalability and efficiency [33,39].

The deliberate restriction of this study to two well-established convolutional architectures was intended to isolate representation-level effects from architectural confounders. VGG16 provides a canonical high-capacity baseline [28], while MobileNetV2 represents an efficient, deployment-oriented design [39]. Excluding deeper residual or compound-scaled networks minimized the influence of advanced architectural mechanisms, enabling clearer attribution of observed improvements to preprocessing choices.

Several limitations warrant consideration. The analysis was confined to two CNN architectures and a binary classification task; future work should examine whether similar trends persist in lesion localization or temporal CEM analysis frameworks [40,41]. More refined anatomical or hybrid representations integrating radiomic or functional parametric features may further enhance performance [6,12,18,21,27]. Clinical implementation will also require continued validation of automated segmentation reliability across heterogeneous acquisition settings. Finally, external validation on multi-center datasets will be essential to confirm generalizability across diverse protocols and populations [19,42].

In summary, this study demonstrates that anatomical breast masking exerts a measurable and architecture-agnostic influence on deep-learning performance in contrast-enhanced mammography. By constraining the input to anatomically relevant regions, it is possible to achieve more stable training, improved discrimination, and enhanced generalization. Although the present analysis focused on binary classification, representation-level stabilization is not inherently task-specific and may extend to multi-class or localization frameworks, where structured input constraints can facilitate feature learning and spatial focus. These findings reinforce the importance of treating preprocessing and input representation as foundational engineering decisions in medical artificial intelligence systems.

Although the results demonstrate consistent improvements across cross-validation folds and an independent hold-out test set, all experiments were conducted on a single publicly available dataset (CDD-CESM, TCIA). Therefore, conclusions regarding robustness across different acquisition protocols, scanners, or patient populations should be interpreted as preliminary. External validation on independent multi-center datasets will be necessary to confirm the generalizability of the proposed preprocessing strategy in heterogeneous clinical environments.

## 5. Conclusions

This study evaluated the impact of anatomical breast masking on deep-learning performance in contrast-enhanced mammography using two standard convolutional neural network architectures, VGG16 and MobileNetV2. The results show that models trained on original images achieve moderate performance and limited generalization, whereas breast-mask-based representations consistently improve discrimination, sensitivity to malignant cases, and training stability across cross-validation folds. These improvements are observed for both high-capacity and lightweight architectures, indicating that the benefit of anatomical masking is largely independent of model complexity.

Overall, the findings highlight image representation design, particularly anatomical constraint, as a key engineering component in medical AI pipelines, offering a simple, computationally neutral, and architecture-agnostic strategy to improve robustness and generalization in contrast-enhanced mammography.

## Figures and Tables

**Table 1:** Performance metrics obtained on the independent hold-out test set for VGG16 and MobileNetV2 under original and breast-mask-based input representations. Accuracy represents the overall proportion of correctly classified cases. AUROC (Area Under the Receiver Operating Characteristic Curve) quantifies threshold-independent discrimination capability. AUPRC (Area Under the Precision–Recall Curve) provides a complementary evaluation under class imbalance conditions. Recall (Malignant) corresponds to sensitivity for malignant cases, while specificity reflects the correct identification of benign/negative cases. Balanced Accuracy is defined as the arithmetic mean of sensitivity and specificity and was used to account for class imbalance.

Model	Input Representation	Accuracy (95% CI)	AUROC (95% CI)	AUPRC (95% CI)	Recall–Malignant (95% CI)	Specificity (95% CI)	Balanced Accuracy (95% CI)
VGG16	Original images	0.705 (0.612–0.789)	0.815 (0.742–0.881)	0.782 (0.704–0.852)	0.608 (0.487–0.719)	0.812 (0.706–0.892)	0.705 (0.622–0.782)
VGG16	Breast mask images	0.774 (0.689–0.848)	0.872 (0.812–0.924)	0.891 (0.834–0.939)	0.726 (0.612–0.823)	0.821 (0.719–0.899)	0.774 (0.694–0.847)
MobileNetV2	Original images	0.651 (0.561–0.733)	0.732 (0.658–0.806)	0.718 (0.639–0.794)	0.602 (0.488–0.712)	0.739 (0.631–0.829)	0.651 (0.569–0.728)
MobileNetV2	Breast mask images	0.712 (0.624–0.791)	0.788 (0.716–0.857)	0.771 (0.694–0.842)	0.641 (0.528–0.748)	0.793 (0.689–0.874)	0.712 (0.631–0.787)

## Data Availability

The raw data for data analysis was uploaded by https://www.cancerimagingarchive.net/collection/cdd-cesm/ (accessed on 1 February 2026). Results findings are reported in the manuscript.
